# Late-Onset Type 1 Diabetes Mellitus and Unexplained Subcutaneous Lesions

**DOI:** 10.7759/cureus.47124

**Published:** 2023-10-16

**Authors:** Malik A Tunc, Karim Amireh, Kevin Brar, Ramyaprabha Bondalapati, Pedro Novo, Alexey Podcheko MD

**Affiliations:** 1 Internal Medicine, American University of the Caribbean, Cupecoy, SXM

**Keywords:** systemic inflammatory and autoimmune disease, insulin autoimmune syndrome, subcutaneous nodule, type 1 diabetes mellitus (t1dm), diabetes, nodular fasciitis

## Abstract

Nodular fasciitis is considered a reactive lesion of connective tissue originating from the proliferation of fibroblasts and myofibroblasts. Nodular fasciitis preponderantly localizes within the higher extremities, trunk, head, and neck. We are presenting a report on the case of a 38-year-old Navy pilot who developed nodular lesions in the area of the sternum and upper back and was diagnosed concomitantly with insulin-dependent diabetes mellitus (type 1 diabetes). The patient was treated for diabetic ketoacidosis using intensive insulin therapy protocol, and the nodules were surgically excised. He was discharged from the hospital four weeks later. In our presentation, we intend to highlight the essential characteristics of this rare lesion through a review of the literature and to identify an attainable link between the development of type 1 diabetes and nodular fasciitis.

## Introduction

Nodular fasciitis is a rapidly growing but benign self-limited myofibroblastic or fibroblastic tumor that typically arises in the upper extremities, trunk, head, and neck. Although the exact etiology of nodular fasciitis remains unknown, it is thought to be a reactive lesion of connective tissue resulting from the proliferation of fibroblasts and myofibroblasts [1-4]. Nodular fasciitis can occur spontaneously or may be preceded by a history of trauma. However, its exact etiology remains unknown. Due to their rapid growth, an abundance of cells, and high level of mitotic activity, these lesions are often misdiagnosed as sarcomas, leading to potential diagnostic challenges and treatment complications [5].

While the relationship between nodular fasciitis and diabetes mellitus (DM) is unclear, a few reports in the literature have discussed the association of inflammatory cutaneous and subcutaneous manifestations with DM [6]. Indeed, according to Horton et al., 79.2% of patients with DM present with some sort of skin disorder. They also insist on the fact that these cutaneous manifestations can be one of the first signs of DM [7]. Dasaclu et al. highlighted that some characteristics of systemic inflammation such as neutrophil-to-lymphocyte ratio or systemic inflammation index, can be directly correlated to one of diabetes' most worrisome complications: retinopathy [8]. As discussed in many of these research papers, one of the common themes with DM is the pro-inflammatory multi-organ processes that can wreak havoc in the patients' bodies. Our report aims to provide further insight into the potential link between nodular fasciitis and type 1 DM (T1DM) and discuss diagnosing, treating, and managing this rare soft tissue tumor.

## Case presentation

We present a case of a 38-year-old male admitted to the hospital with complaints of nausea, weakness, thirst, frequent urination, headache, and pain in the sternum and back of his neck. The patient reported that he had been getting annual physical exams for the past 15 years and had no history of any chronic illness. However, he reported being stressed because of a recent divorce about four months ago, and he had flu-like symptoms about three months ago. Two weeks before presenting to the hospital, the patient noticed two nodules on his chest and one on his back, all 2x2 cm in size. Around the same time, he noticed that he drank more water than usual, accompanied by polyuria. The patient did not have a history of hypertension, but his blood pressure was 156/111 mmHg on admission for his aforementioned symptoms, which was later stabilized to 125/80 mmHg. The patient's family history revealed that his mother was alive and healthy, while his father died at 72 from colon cancer. The patient had been a Navy pilot for the past 15 years.

Upon admission, the following laboratory tests were ordered because of the patient's symptoms of polydipsia, polyuria, nausea, weakness, and headache, which were all presumptive of hyperglycemia-induced ketoacidosis: complete blood count, arterial blood gas, biochemistry panel, urinalysis, and EKG. Additionally, coagulation studies, an inflammatory panel, and an immunologic panel were also ordered because of the patient's flu-like symptoms and subcutaneous nodules (Table 1).

**Table 1:** Remarkable laboratory results. Only remarkable laboratory findings are included and, hence, only pertinent positives and pertinent negatives.

Laboratory Test	Value	Reference Range
WBC cell count	21,100 /mm^3^	4,500-11,000 /mm^3^
Neutrophil %	23%	54-62%
Lymphocyte %	72%	25-33%
pH	7.06	7.35-7.45
CO2	33.5	45-45
HCO3	20.2	22-26
Blood glucose	220 mg/dL	<120mg/dL
Blood lactate	3.2mmol/L	0.5-1.0mmol/L
Blood urea nitrogen	12 mmol/L	2.1 to 8.5 mmol/L
Potassium	5.4 mmol/L	3.6-5.2 mmol/L
Chloride	112 mmol/L	96-106 mmol/L
Creatinine	136umol/L	65-113 umol/L
HbA1c	15.2 %	<6%
C-peptide	56.2 pmol/L	370-1470pmol/L
GAD (anti-glutamic acid decarboxylase) antibody titer	2.8	0-1.0
CRP	positive	negative
ESR	45mm	<15mm
Urinalysis		
Glucose	4+	negative
Ketones	3+	negative
Protein	2+	negative

The lab results showed that the patient had lymphocytosis and positive inflammatory markers, which suggested an inflammatory or immunological reaction. These results are a common occurrence in diabetic ketoacidosis (DKA). The patient also had very high HbA1c levels, and ketones in the urine were further indications of DKA. The high HbA1c levels indicated that the patient had been living with uncontrolled diabetes for an extended period. Simultaneously, ketones in the urine and reduced levels of C-peptide were clear signs of insulin deficiency.

Additionally, the patient's labs showed hyperkalemia and hyperchloremia, likely because of dehydration and reduced kidney function caused by nephrotic syndrome as highlighted by the protein in the urine. This condition can cause kidney damage and lead to electrolyte imbalances. Interestingly, the onset of diabetic nephropathy depends on the type. In T1DM, diabetic kidney disease usually occurs approximately 10 years after the diagnosis, while in type 2 diabetes mellitus (T2DM), it may be present at the time of diagnosis [9]. This indicates that our patient did not follow this trend. The etiology of the nodules was uncertain at this time. However, an autoimmune reaction was suspected in association with the onset of T1DM, an autoimmune disorder characterized by the destruction of pancreatic beta cells, resulting in insulin deficiency. The diagnosis of DM involves repeatedly fasting high blood glucose readings. Furthermore, to define it as type 1, different studies can be conducted; however, the two most common are the C-peptide levels and antibodies against glutamic acid decarboxylase (GAD), which is responsible for the conversion of glutamic acid to GABA. If C-peptide levels are elevated, it indicates insulin resistance, hence T2DM; however, if C-peptide levels are reduced, it indicates insulin insufficiency, hence type 1 DM (T1DM). GAD antibody positivity indicates some inflammatory process in the pancreas supporting a diagnosis of T1DM [10]. Our patient laboratory results, elevated blood glucose, reduced C-peptide levels, and positive GAD antibody, confirm the suspected diagnosis of T1DM based on the patient's clinical manifestations.

The medical team's treatment plan for the patient's diabetic ketoacidosis (DKA) appeared to align with the standard guidelines for DKA management. The mainstay of treatment for DKA consists of IV fluid resuscitation, electrolyte repletion especially potassium, and insulin therapy. Isotonic saline solution was given as well as insulin. Since his blood potassium level was slightly elevated, there was only a mild risk for hypokalemia following insulin administration because of insulin's effect of enabling potassium into adipose and muscle cells. The team prescribed subcutaneous insulin isophane and subcutaneous neutral protamine Hagedorn insulin (insulin NPH) to address the patient's insulin deficiency. Insulin isophane is an intermediate-acting insulin that helps control blood glucose levels over extended periods, in-between meals. Insulin NPH is a short-acting insulin that helps control blood glucose levels immediately after meals by being given before meals.

**Figure 1:**
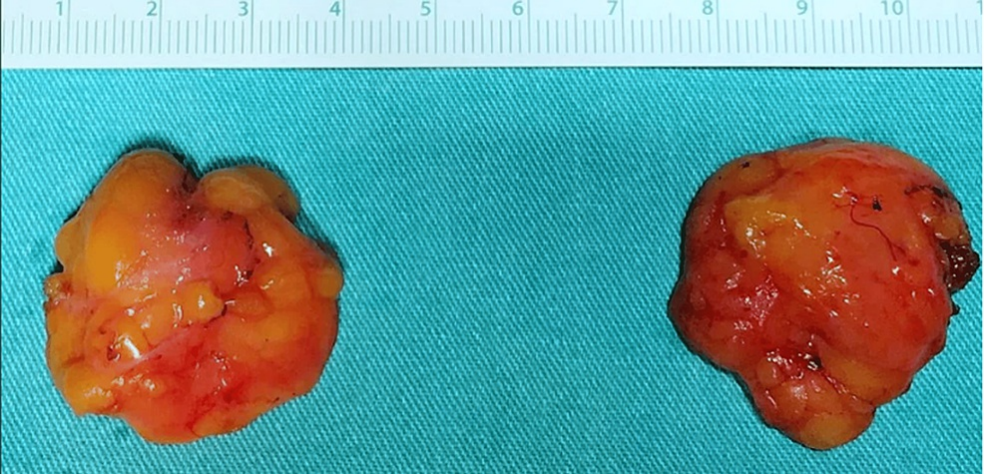
Removed nodules demonstrate a relatively well-circumscribed lesion, without a clear capsule.

**Figure 2:**
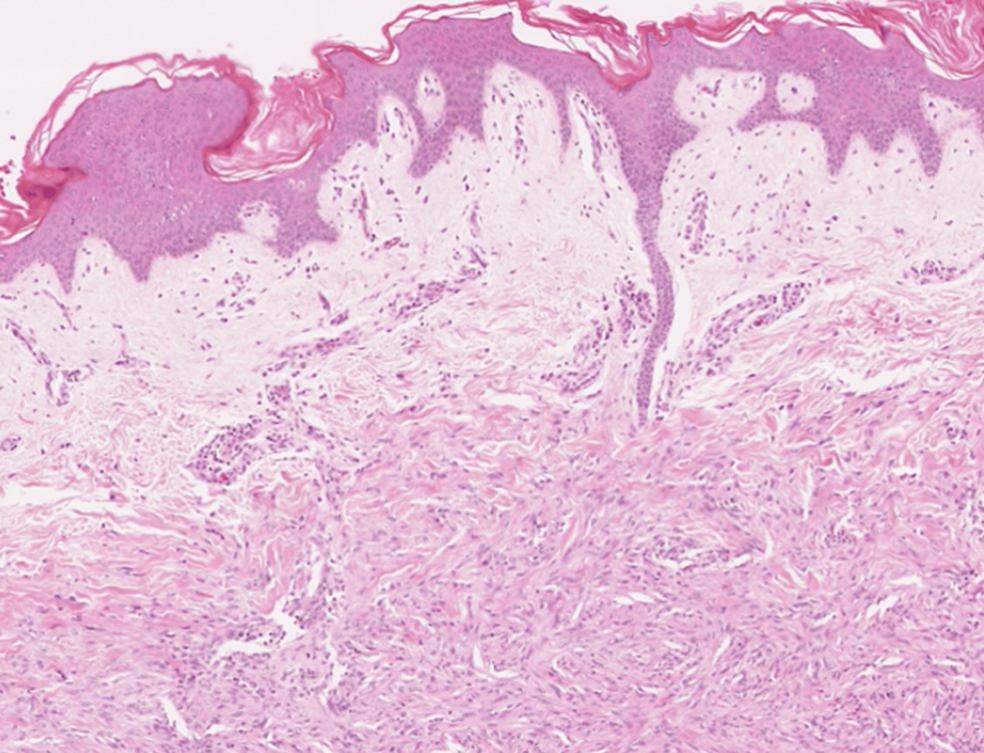
Histopathological examination (HE stain, 20x magnification): The subcutaneous connective tissue appears to be infiltrated by a well-circumscribed nodular proliferation. Marginal areas presented more fibrous stroma, and the proliferating cells closely resembled immature fibroblasts and histiocytes of granulation tissue.

**Figure 3:**
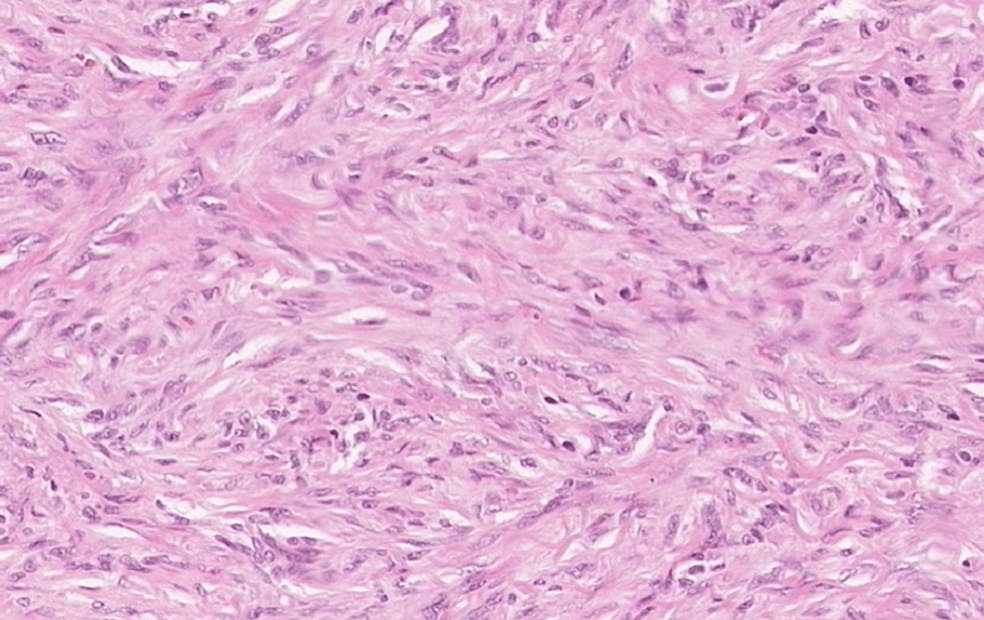
Histopathological examination (HE stain, 40x magnification): The subcutaneous connective tissue appears to be infiltrated by a well-circumscribed nodular proliferation. Marginal areas presented more fibrous stroma, and the proliferating cells closely resembled immature fibroblasts and histiocytes of granulation tissue.

**Figure 4:**
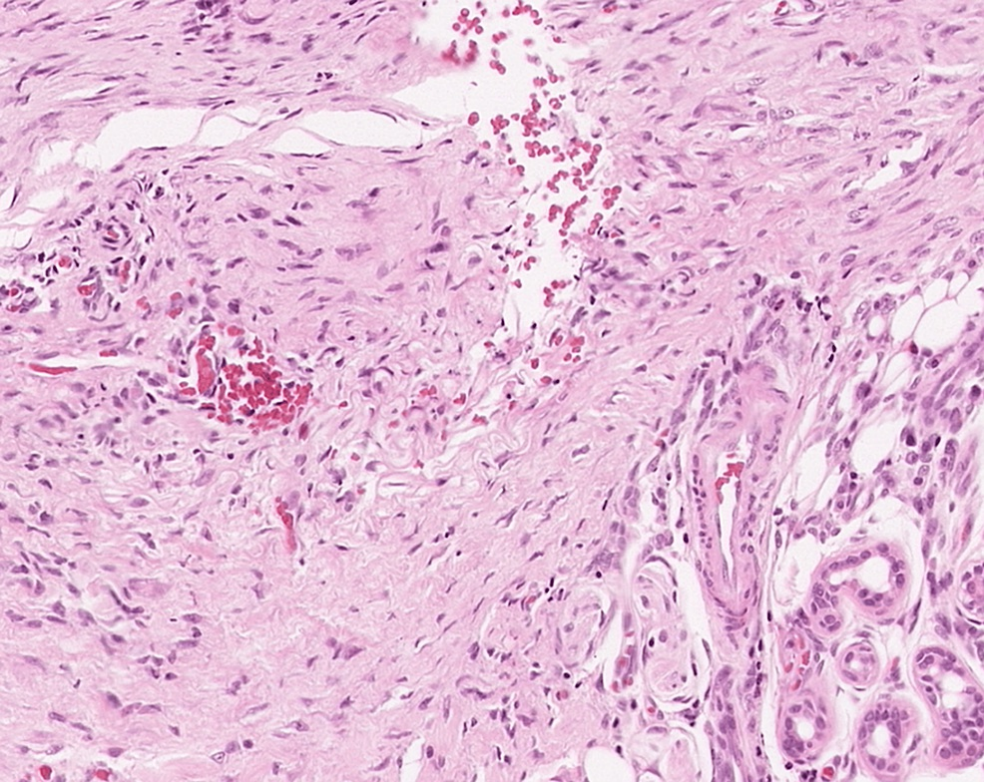
Histopathological examination (HE stain, 40x magnification): The subcutaneous connective tissue appears to be infiltrated by a well-circumscribed nodular proliferation. Marginal areas presented more fibrous stroma, and the proliferating cells closely resembled immature fibroblasts and histiocytes of granulation tissue.

At admission, a biopsy of the nodules was sent to the pathology department; however, due to a technical issue, it took 10 days for the results to be released to the medical team. The results ruled out malignancy and cleared the nodules for removal by excision. Finally, 10 days after admission and stabilization of the patient's symptoms, the team conducted a removal of the nodules under local anesthesia after an initial ultrasound. The sonography showed within the fascia of the left pectoralis major muscle a nodular mass of 23×20×24 mm and within a superficial layer of the trapezius muscle of the left a nodular mass of 15×21×17 mm. No signs of invasions of surrounding structures nor abnormal lymph nodes were found in the explored region. These findings indicated low potential for cancerous masses and that this patient's nodules could be safely removed by surgery. After the nodules were excised, a biopsy and a histopathological examination were performed. The removed nodules demonstrated a relatively well-circumscribed lesion without a clear capsule, which looked similar to a lipoma (Figure 1). The histopathological examination revealed that the subcutaneous connective tissue was infiltrated by well-circumscribed nodular proliferation. Marginal areas presented fibrous stroma, and the proliferating cells closely resembled immature fibroblasts and histiocytes of granulation tissue (Figures 2-4). The surgical wounds healed by primary intention, and the sutures were removed eight days after the procedure. The patient was discharged three weeks after admission in a good state. Patients with DKA get discharged three to five days after admission on average [11]. However, while being hospitalized, the patient was enrolled in a clinical trial that was running in the hospital as a newly diagnosed T1DM patient. The study aimed to monitor his immune responses to insulin therapy. No conclusions were drawn from this clinical trial.

## Discussion

This case report discusses a patient who presented with DKA because of insulin deficiency caused by destroying beta cells in the pancreas, a hallmark of T1DM. T1DM is an autoimmune disorder that affects approximately 5-10% of people with diabetes, and it is characterized by the gradual destruction of pancreatic beta cells, leading to insulin deficiency. The average age of onset of T1DM is typically in the range of 11-14 years old, and it is considered to be an autoimmune response. The genetic susceptibility of HLA-DR3 or HLA-DR4 is found in 90-95% of Caucasians, and environmental factors such as viral infections (rubella, mumps, coxsackie, EBV, hepatitis B) can contribute to the onset of T1DM [12]. Beta cells show histological evidence of damage, and autoantibodies are typically present when the initial diagnosis of hyperglycemia is made. β cell mass declines over time, and insulin secretion becomes impaired. The rate of decline varies from individual to individual. Patients with T1DM are prone to developing ketoacidosis without insulin treatment, requiring exogenous insulin for survival.

The differential diagnosis for a 38-year-old navy pilot who suddenly developed a nodular lesion in the area of the sternum and at the same time was diagnosed with insulin-dependent T1DM included various reactive and neoplastic lesions. Given the rapid onset of the patient’s swelling, malignant lesions were considered more likely than benign lesions. Grossly, nodular fasciitis may be soft and gelatinous in consistency, similar to a myxoma or a benign peripheral nerve sheath tumor [13]. When more collagenous and firm, it may resemble fibromatosis, a locally recurring, non-metastasizing proliferation of fibrous tissue. A few malignant neoplasms can share histologic features with nodular fasciitis. Fibrosarcomas have an active mitotic rate like nodular fasciitis, but they are characterized by atypical mitoses and significant nuclear pleomorphism [14].

Additionally, it can be differentiated by its growth pattern and cellularity. Fibrosarcomas are characterized by a herringbone pattern filled with spindle cells. Fibrosarcomas typically occur in a deeper location, are larger, and have a longer duration [15]. Histologically, the differential diagnosis may include proliferative fasciitis, a variant of nodular fasciitis, and can also contain fibroblasts and myofibroblasts. It also proliferates, and a history of trauma is reported in approximately one-third of cases. It tends to be poorly circumscribed, and it often contains large, polygonal “ganglion-like” cells. It also affects a slightly older population (age 40-70) [16].

Nodular fasciitis is a benign, self-limited neoplasm of fibroblastic and myofibroblastic derivation. It usually presents as a small but rapidly growing mass commonly seen in the upper extremities, head, neck, and trunk, although it can occur almost anywhere, including superficial growth. Nodular fasciitis is most commonly seen in individuals between 20-40 years of age and is equally prevalent in males and females. Despite its benign nature, nodular fasciitis can often be misdiagnosed as sarcoma. However, in an extensive series of 895 cases of nodular fasciitis, only 1% of cases recurred [17]. Nodular fasciitis was previously speculated to arise because of a reactive process, with 10-15% of patients reporting a history of trauma. However, recent research has suggested that nodular fasciitis is driven by the overexpression of the USP6 gene caused by a balanced rearrangement involving the USP6 gene. Most of the fusion products in nodular fasciitis are MYH9-USP6 [18]. This discovery has allowed for a better understanding of the pathophysiology of nodular fasciitis, which will facilitate the development of more effective and targeted treatment options.

To establish a differential diagnosis, nodular fasciitis cells show muscle-specific actin (MSA) and smooth muscle actin (SMA) positivity [19]. It is vital to include nodular fasciitis in the differential diagnosis when presented with a rapidly growing ulcerated lesion as it can simulate malignancy both clinically and histologically. In a study of 53 cases of nodular fasciitis, only 23 cases (43%) were correctly diagnosed, while sarcoma was diagnosed in 11 (21%) [20]. Therefore, correctly diagnosing nodular fasciitis is pivotal in preventing its overtreatment as a more aggressive and malignant growth. This highlights the importance of considering nodular fasciitis in diagnosing rapidly growing ulcerated lesions.

The relationship between T1DM and nodular fasciitis remains unknown, although the rapidly evolving status of nodular fasciitis indicates a possible trigger reaction similar to T1DM. Patients with T1DM are known to be at increased risk of developing other autoimmune diseases, most commonly autoimmune thyroiditis and celiac disease. Furthermore, one-quarter of T1DM patients younger than 21 have one or more other organ-specific antibodies [20]. While there is currently no reported association between these two diagnoses in the literature, a retrospective case series may be an excellent option for exploring the possible correlation. The proposed hypothesis suggests that there may be a genetic predisposition to developing nodular fasciitis. This genetic susceptibility may be triggered by an infection that leads to lymphocytic proliferation and subsequent infiltration of subdermal tissue. A possible mutation in the targeted tissue may be involved in the lymphocytic infiltration, leading to an inflammatory reaction. This inflammation, in turn, may cause fibroblast proliferation and histiocyte activation, leading to the development of nodular fasciitis. Further research is needed to determine the validity of this hypothesis and the underlying mechanisms involved in the pathogenesis of nodular fasciitis.

## Conclusions

In conclusion, this case report highlights the importance of considering the correlation between T1DM and nodular fasciitis. The etiology of nodules in this patient is uncertain, but an autoimmune reaction is suspected to be associated with the onset of T1DM. Prompt insulin therapy and aggressive management of diabetic ketoacidosis stabilized the patient's symptoms, allowing for the successful removal of nodules. While the relationship between nodular fasciitis and T1DM is unknown, further exploration through a retrospective case series could highlight possible correlations between the two diagnoses.
